# Beyond the Acne

**DOI:** 10.7759/cureus.40897

**Published:** 2023-06-24

**Authors:** Manush Sondhi, Warda Maqsood, Sarwat Umer

**Affiliations:** 1 Internal Medicine, Louisiana State University Health Sciences Center, Shreveport, USA; 2 Rheumatology, Louisiana State University Health Sciences Center, Shreveport, USA

**Keywords:** adalimumab, pustulosis, sacroiliitis, sapho, acne

## Abstract

Synovitis, acne, pustulosis, hyperostosis, and osteitis (SAPHO) is a relatively rare and often underdiagnosed disorder characterized by chronic inflammation affecting the bones, joints, and skin. While the precise cause of SAPHO syndrome remains elusive, multiple factors such as genetics, immunological dysregulation, and bacterial influences have been implicated in its pathogenesis. One notable aspect of SAPHO syndrome is the wide variability of symptoms experienced by afflicted individuals. A diverse array of osteoarticular manifestations may be observed, with common sites of involvement including the anterior chest wall, sacroiliac joints, and peripheral joints. Concurrently, patients often present with various skin disorders, such as palmoplantar pustulosis or acne, further adding to the complexity of the syndrome's clinical presentation. Treatment strategies for SAPHO syndrome primarily focus on managing symptoms and improving the quality of life for affected individuals. Nonsteroidal anti-inflammatory drugs (NSAIDs), corticosteroids, methotrexate (MTX), and tumor necrosis factor (TNF) inhibitors are considered to modulate the immune response and provide relief. One of the challenges encountered in diagnosing SAPHO syndrome is its potential overlap with other related conditions, leading to diagnostic confusion and difficulties. Distinguishing SAPHO syndrome from similar entities can be complex, requiring a comprehensive evaluation of clinical features, imaging studies, and laboratory investigations. We would like to share an intriguing case involving a 28-year-old woman who arrived with perplexing symptoms of pain in her bilateral hands and feet, her lower back, and acne in the bilateral upper arms and thighs. Through a comprehensive workup, the underlying SAPHO syndrome was uncovered, and it was effectively managed using adalimumab.

## Introduction

Synovitis, acne, pustulosis, hyperostosis, and osteitis (SAPHO), a chronic inflammatory disease, is also labeled as sternocostoclavicular hyperostosis, acne-associated spondyloarthropathy, and pustulotic arthro-osteitis [1]. Chronic recurrent multifocal osteomyelitis (CRMO) is a disease of children with similar features with axial or appendicular joint involvement but with or without skin disease. SAPHO syndrome is a rare disease, almost found in 1 in 10,000 white populations. Individuals between the ages of 30 and 50 are predominantly affected. There is an apparent female predominance, particularly among patients less than 30 years of age at the onset [2]. Genetic, immunological, and bacterial (association with Propionibacterium acnes) contribute to disease development [3]. Bone involvement appears radiologically as osteosclerosis, with cortical thickening, and most commonly affects the anterior chest wall, thoracic and lumbar spine, and sacroiliac joint. Skin findings in patients with SAPHO syndrome include a variety of acneiform and neutrophilic dermatoses. SAPHO syndrome can exhibit overlapping features with conditions such as rheumatoid arthritis, psoriatic arthritis, ankylosing spondylitis, osteomyelitis, and bone tumors. Therefore, it is crucial to thoroughly evaluate and consider other potential differential diagnoses when encountering this syndrome. We present a fascinating case involving a 28-year-old woman with interesting findings who was diagnosed with SAPHO syndrome.

## Case presentation

**Figure 1:**
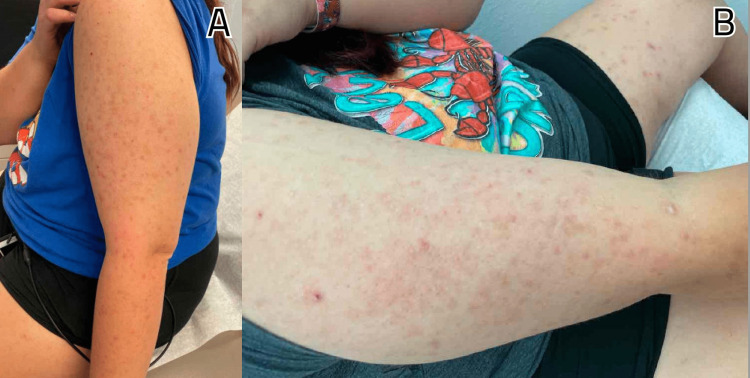
A and B depict papules, nodules, and pustules scattered in the bilateral arms.

**Figure 2:**
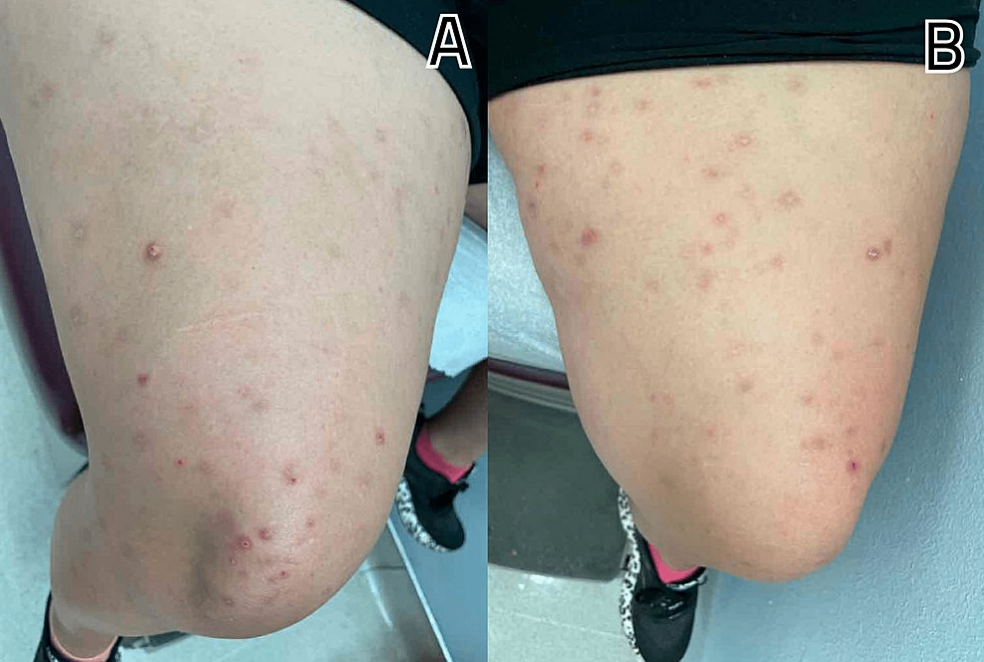
A and B depict papules, nodules, and pustules scattered in the bilateral thighs.

A 28-year-old Caucasian female with a history of pre-diabetes, diagnosed for the last two months and currently on metformin 500 mg twice daily, presented to the clinic with intermittent pain in both hands and feet over the past year, along with recurrent episodes of lower back pain for the last two years. She reported occasional mild joint swelling in her hands and ankles but no recorded instances of joint pain or stiffness. The patient mentioned that the pain showed no diurnal variation or correlation with increased activity. Initially, she took ibuprofen intermittently, but eventually, the pain worsened, leading to a referral to rheumatology. Additionally, she complained of acne on her bilateral upper arms (Figures 1A, 1B) and thighs (Figures 2A, 2B), accompanied by itching, which had persisted for the past four years. However, she did not have a history of facial acne. Seeking assistance, she visited a dermatologist who prescribed minocycline 100 mg twice daily and salicylic lotion but did not experience any relief. The patient had been suffering from chronic diarrhea for the past 10 years, with intermittent episodes of bloody stools. She underwent a colonoscopy twice, which revealed normal results, leading to a diagnosis of irritable bowel syndrome.

Furthermore, she had undergone surgery to remove a posterior calcaneal exostosis in her right heel two years ago. There was no history of nail or scalp lesions, enthesitis, fever, photosensitive rashes, morning stiffness, alopecia, miscarriages, weight changes, mucosal ulcers, chest pain, dyspnea, cough, abdominal pain, muscle pain or weakness, numbness, dry eyes, dry mouth, or any other eye complaints. The patient had no significant family history, and there was no history of smoking, alcohol consumption, or recreational drug use. During a physical examination, papules, nodules, and pustules were observed scattered on the bilateral arms and thighs. Tenderness was noted upon palpation of the lower back.

**Figure 3:**
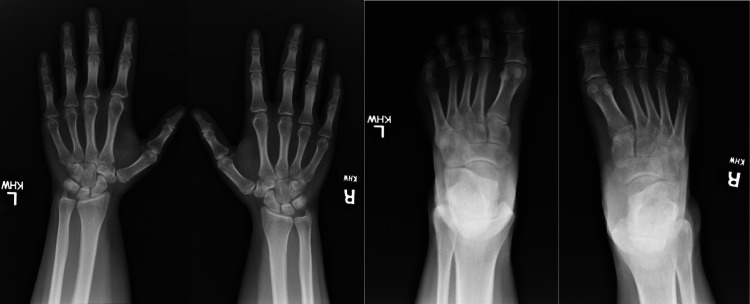
Radiography of the hands and feet shows no acute fracture, dislocation, definite erosion, or significant degenerative changes. Diffuse osteopenia on the right foot with severe tarsometatarsal joint space narrowing is observed.

**Figure 4:**
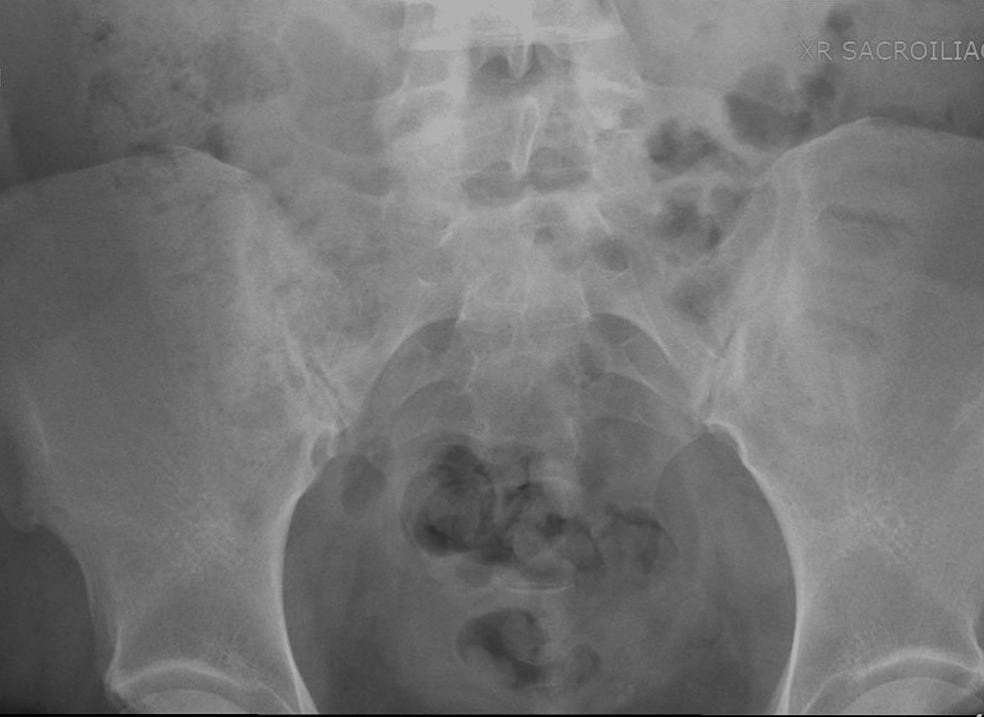
Radiography of the lumbar spine and sacroiliac joints shows mild sclerosis of the sacroiliac joints.

**Figure 5:**
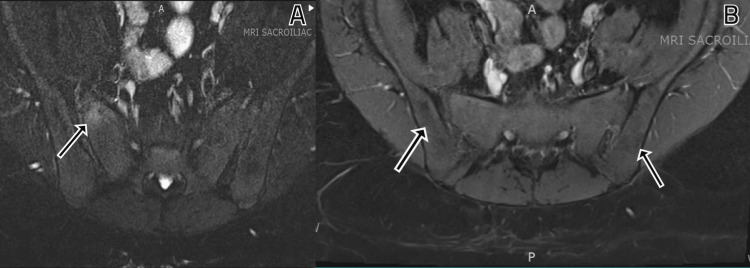
MRI of the sacroiliac joints. 5A shows foci of abnormal signal intensity in the right ischium marked by the arrow hypointense on T1 and hyperintense on STIR images. 5B depicts foci of bone marrow edema marked by the arrows in the bilateral SI joints pointing towards bilateral sacroiliitis.

Blood count, blood chemistry, thyroid stimulating hormone, total testosterone, and dehydroepiandrosterone levels were within normal range. However, laboratory investigations revealed an increased erythrocyte sedimentation rate (ESR) of 32 mm/hr (normal value 0-20 mm/hr), C-reactive protein (CRP) level of 1.52 mg/dl (normal value 0.00-0.90 mg/dL), and complement component 3 level of 201 mg/dl (normal value 50-180 mg/dL). Immunological tests, including rheumatoid factor, anti-citrullinated cyclic peptide antibody, anti-nuclear antibody, anti-Sjögren's-syndrome-related antigen A and B, and human leukocyte antigen B27 (HLA-B27), were negative. X-rays of the hands did not reveal any acute fractures, dislocations, or significant soft tissue swelling or erosions. X-rays of the feet showed diffuse osteopenia on the right foot and severe tarsometatarsal joint space narrowing bilaterally but no definitive erosions or significant degenerative changes (Figure 3). To evaluate for spondyloarthropathy, X-rays of the lumbar spine and sacroiliac (SI) joints were performed, indicating mild sclerosis of the SI joints (Figure 4). Further magnetic resonance imaging of the sacroiliac joints demonstrated areas of bone marrow edema in the bilateral SI joints, resorption of the subchondral plate on the right with erosions, and multiple foci of abnormal signal intensity in the right ischium (hypointense on T1 and hyperintense on STIR images). These findings were consistent with bilateral sacroiliitis, multifocal signal abnormality in the right ischium, and mild bone expansion (Figures 5A, 5B).

**Figure 6:**
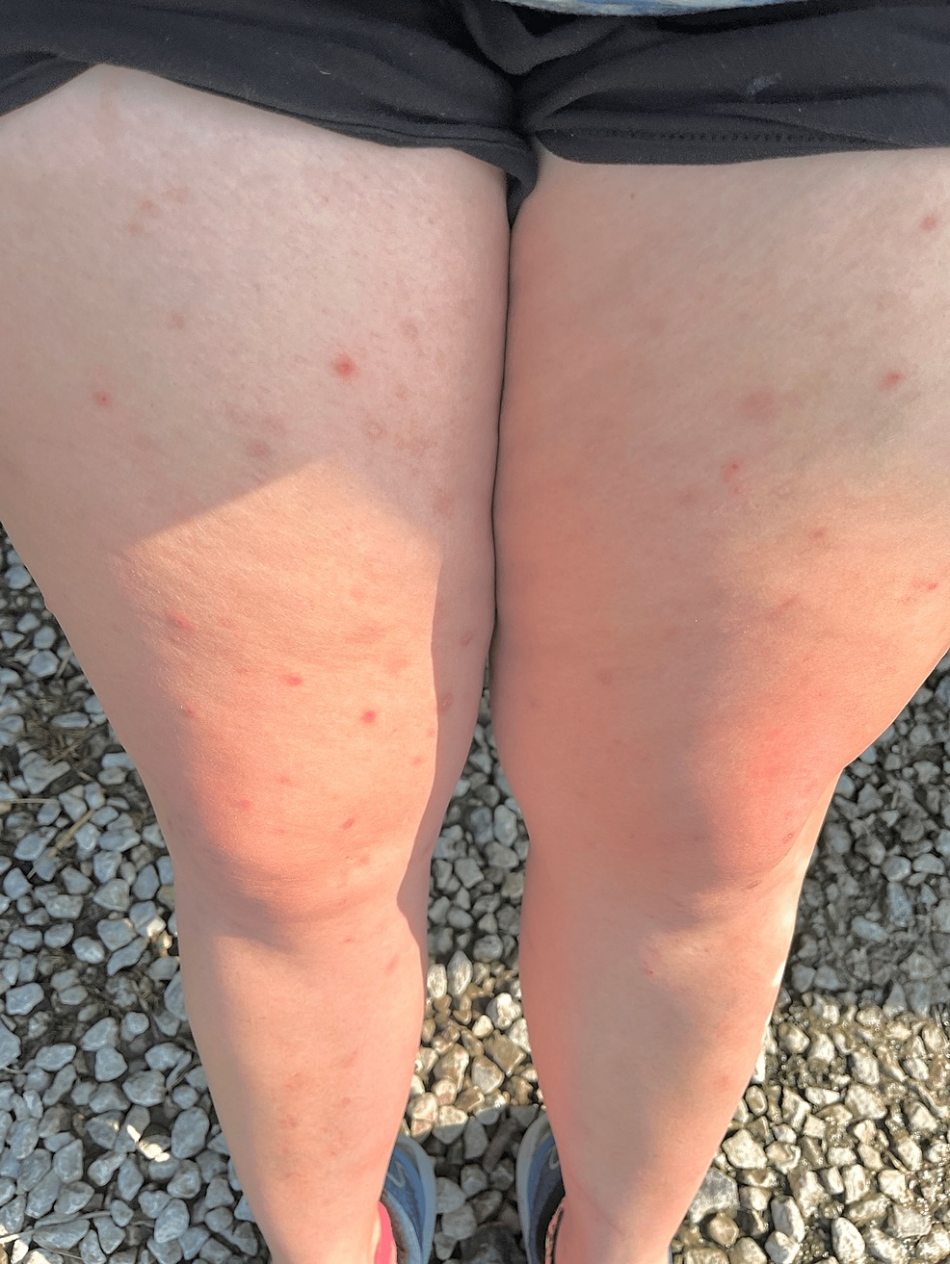
Significant improvement in the papules, nodules, and pustules scattered across both thighs upon initiating treatment with adalimumab and over-the-counter products containing benzoyl peroxide.

The diagnosis of SAPHO syndrome was made based on the involvement of the SI joints associated with severe acne and pustulosis, following the modified Kahn criteria. The patient was initiated on subcutaneous adalimumab at a dose of 40 mg every 14 days, along with over-the-counter products containing benzoyl peroxide. She reported significant improvement in acne and pain in her extremities and back over 8 months (Figure 6).

## Discussion

SAPHO syndrome, an autoinflammatory disorder primarily affecting adults, bears a striking resemblance to CRMO observed in children. SAPHO syndrome encompasses a broad spectrum of anomalies characterized by variable combinations of osteoarticular and cutaneous manifestations of varying degrees of severity. Osteoarticular manifestations usually include synovitis, osteitis, hyperostosis, or enthesitis. In 65 to 90% of patients, it affects the anterior chest wall, including the sternocostal, sternoclavicular joints, and the costoclavicular ligament. In 32 to 52% of patients, it involves the SI joint and spine, which is often unilateral and primarily due to osteitis, although synovitis may occur secondarily. In less than 30% of patients, peripheral joints are involved, affecting the hip, knee, and ankle joints more commonly involved than upper-extremity joints. Skin findings in patients with SAPHO syndrome include a variety of acneiform and neutrophilic dermatoses [4]. Palmoplantar pustulosis is the most common manifestation, observed in up to 60% of patients, followed by nodulocystic acne, often resulting in residual scarring seen in 25% of patients [5]. Other features of follicular occlusion syndromes may be present, including hidradenitis suppurativa [6]. Inflammatory bowel disease is reported in up to 10% of patients with SAPHO syndrome, with Crohn's disease being more frequently reported than ulcerative colitis [7].

Nonspecific inflammatory changes may be observed, including elevations in the ESR and CRP, elevated complement levels, mild leukocytosis, and thrombocytosis, but they are not universal. In cases of SAPHO, there is no notable elevation in the frequency of HLA-B27. SAPHO syndrome should be suspected in patients with bone and joint symptoms consistent with inflammatory arthritis or osteitis, especially when involving the anterior chest wall, SI joints, or spine, and particularly when associated with a neutrophilic dermatosis or acneiform eruption [8]. Standard imaging modalities, such as X-rays, MRIs, and bone scans, are routinely conducted for diagnostic purposes. MRI detects osteitis not visible on plain radiographs and, in addition, provides soft tissue data. The bull's head sign (or lobster claw sign) on bone scan imaging is characteristic of sternoclavicular involvement [9]. Kahn et al.'s proposal in 1994, modified in 2003, is the most commonly used diagnostic criteria for this syndrome [8]. The Kahn criteria for diagnosing SAPHO syndrome include inclusion criteria such as bone-joint involvement associated with palmoplantar pustulosis and psoriasis vulgaris, bone-joint involvement associated with severe acne, isolated sterile hyperostosis/osteitis (adults), chronic recurrent multifocal osteomyelitis (children), and bone-joint involvement associated with chronic bowel diseases. Infectious osteitis, tumoral conditions of the bone, and noninflammatory condensing lesions of the bone must be excluded. A diagnosis of SAPHO is considered in patients displaying any one of the inclusion criteria. It is also crucial to exclude other rheumatic and autoinflammatory disorders affecting the bones and joints, such as rheumatoid arthritis, axial spondylarthritis, and psoriatic arthritis, making it a diagnosis of exclusion. Unlike SAPHO, osteomyelitis is less likely to be multifocal, and osteosarcoma typically occurs as a single bone lesion, often affecting the long bones and having distinct radiographic features. A bone biopsy can establish a definitive diagnosis.

Current treatments aim to relieve pain and include NSAIDs as first-line agents. Systemic corticosteroids, disease-modifying anti-rheumatic drugs, and biologics targeting TNF-alpha have benefited patients. In patients with only bone and joint disease with an inadequate response after four weeks of treatment with at least two different NSAIDs or short-term glucocorticoids, the treatment approach depends on the pattern of joint involvement. For peripheral arthritis without axial disease, oral MTX 15 to 25 mg once weekly is preferred. However, if there is an inadequate response to MTX after three months, switching to a TNF inhibitor or continuing MTX and adding a TNF inhibitor is preferable. A TNF inhibitor is preferred in cases of severe enthesitis with axial disease. Monoclonal antibodies are preferred for psoriatic skin disease, uveitis, or other manifestations. Oral retinoids are the first line of therapy for palmoplantar pustulosis, while antibiotics with anti-inflammatory properties help improve both skin and bone symptoms in cases of acne.

In our patient, although there were no chest wall complaints, and the chest X-ray was normal, the SI joint was involved, which is the second most common osteoarticular manifestation. Sacroiliitis, along with the presence of severe acne, fulfills the inclusion criteria for SAPHO syndrome, according to Kahn et al.'s proposal in 2003 [9]. The exclusion criteria, including infectious osteitis, bone tumors, and noninflammatory bone lesions, were ruled out by MRI. Additionally, the patient developed posterior calcaneal exostosis two years ago, which interestingly shares a resemblance to the excessive bone growth observed in SAPHO syndrome. The absence of HLA-B27 positivity, predominant unilateral sacroiliitis, and the absence of additional symptoms such as uveitis, dactylitis, enthesitis, and psoriatic rash aid in distinguishing SAPHO syndrome in this particular case from other potential differential diagnoses. As axial involvement in the form of sacroiliitis was prominent, the patient was started on subcutaneous adalimumab at a dose of 40 mg every 14 days, along with over-the-counter products containing benzoyl peroxide. After approximately eight months of initiating the treatment, she reported a significant improvement in her acne and the pain experienced in her extremities and back.

Diagnosing SAPHO syndrome can be challenging. The clinical manifestations and radiological features of this syndrome can vary widely and may not align with well-defined disease entities. Additionally, the absence of concurrent clinical signs can further complicate the correlation and potentially result in delayed diagnosis. It often requires a multidisciplinary approach due to its diverse manifestations involving multiple systems.

## Conclusions

In conclusion, SAPHO syndrome is an uncommon inflammatory syndrome characterized by a diverse range of osteoarticular and cutaneous manifestations. The complexity of its clinical presentation can make diagnosis challenging, leading to underdiagnosis in many cases. However, early recognition of SAPHO syndrome is essential to avoid unnecessary treatments, such as emollients, long-term antibiotic use, or invasive procedures. By increasing awareness among healthcare professionals and adopting a multidisciplinary approach, we can improve the diagnosis and management of SAPHO syndrome, ultimately providing appropriate and timely care to affected individuals.
